# Reply to Omansen, T.F.; Ramharter, M. Intensive Therapeutic Plasma Exchange for Severe Yellow Fever: What Is the Evidence? Comment on “Ho et al. Intensive Therapeutic Plasma Exchange—New Approach to Treat and Rescue Patients with Severe Form of Yellow Fever. *Trop. Med. Infect. Dis.* 2025, *10*, 39”

**DOI:** 10.3390/tropicalmed10120343

**Published:** 2025-12-08

**Authors:** Yeh-Li Ho, Youko Nukui, Paula Ribeiro Villaça, Erica Okazaki, Nelson Hidekazu Tatsui, Lucas Chaves Netto, Daniel Joelsons, Tania Rubia Flores da Rocha, Fernanda de Mello Malta, João Renato Rebello Pinho, Aluisio Augusto Cotrim Segurado, Vanderson Rocha

**Affiliations:** 1 Departamento de Infectologia e Medicina Tropical, Hospital das Clinicas, Faculdade de Medicina, Universidade de Sao Paulo, Sao Paulo 05403-000, Brazil; 2 Serviço de Hematologia, Hemoterapia e Terapia Celular, Hospital das Clinicas, Faculdade de Medicina, Universidade de Sao Paulo, Sao Paulo 05403-000, Brazil; 3 LIM07, Departamento de Gastroenterologia, Hospital das Clinicas, Faculdade de Medicina, Universidade de Sao Paulo, Sao Paulo 05403-000, Brazil; 4 Fundacao Pro-Sangue, Sao Paulo 05403-000, Brazil; 5 Churchill Hospital, Oxford University Hospitals, Oxford OX3 7LE, UK

Dear Drs. Omansen and Ramharter, we appreciate your thoughtful comments regarding our experience of intensive therapeutic plasma exchange for severe yellow fever [1]. We agree that randomized clinical trials (RCTs) are the gold standard for generating scientific evidence to inform healthcare decision-making. However, it is important to note that RCTs are only feasible when there is an established treatment that modifies the natural course of a disease, allowing for a comparative analysis to demonstrate, at the very least, non-inferiority. To the best of our knowledge, at the time of our article’s publication, there were no reports of therapeutic measures that modify the lethality of severe yellow fever. Even in liver transplant cases, the survival rate was 42.8%, which is lower than that observed with our intervention [2].

Regarding the possibility of patient selection bias, all of our patients met the criteria for severe yellow fever as defined by the Brazilian Ministry of Health and also fulfilled the Clichy criteria [3,4], widely used for liver transplantation in cases of fulminant hepatitis—further reinforcing the severity of their condition. Our approach aims to halt clinical deterioration in patients with severe yellow fever and prevent fatal outcomes. We agree that this strategy represents an initial step toward reducing the mortality of severe yellow fever cases and should be tested in other centers, ideally through a randomized clinical trial, if it is feasible.

As for the improvements in supportive care in intensive care units, we certainly learned valuable lessons throughout the outbreak. However, these improvements were the most significant in the early weeks, when patients in Group 1 predominated. Supportive therapies in Groups 2 and 3 were similar.

Finally, regarding albumin-based dialysis systems, our experience with MARS was not favorable, and these patients ultimately had fatal outcomes. In our understanding, considering the multiple mechanisms involved in bleeding in yellow fever, although MARS has the potential to remove toxins and restore metabolic balance, it may not able to restore hemostasis [5,6]. As consequence, patients still require massive transfusions of plasma and cryoprecipitate to correct coagulation disorders. Nonetheless, we believe that MARS is worth testing in centers where the necessary equipment is available, in order to compare its results with those of intensive therapeutic plasma exchange.

## References

[1] Omansen T.F., Ramharter M. (2025). Intensive Therapeutic Plasma Exchange for Severe Yellow Fever: What Is the Evidence? Comment on Ho et al. Intensive Therapeutic Plasma Exchange—New Approach to Treat and Rescue Patients with Severe Form of Yellow Fever. *Trop. Med. Infect. Dis.* 2025, *10*, 39. Trop. Med. Infect. Dis..

[2] Song A., Abdala E., Waisberg D., Martino R.B., Li H.Y., Malbouisson L.M.S., Tanigawa R.Y., Neto A.D., Andrade G.M., Ducatti L. (2018). Transplante de fígado em casos graves de febre amarela: A experiência do hcfmusp. Braz. J. Infect. Diseases.

[3] Ministerio da Saude—Secretaria de Vigilancie em Saude, Departmento de Imunizacao e Doencas Transmissiveis (2020). Manual de Manejo Clínico da Febre Amarela.

[4] Bernuau J., Goudeau A., Poynard T., Dubois F., Lesage G., Yvonnet B., Degott C., Bezeaud A., Rueff B., Benhamou J.P. (1986). Multivariate analysis of prognostic factors in fulminant hepatitis B. Hepatology.

[5] Novelli G., Rossi M., Pretagostini R., Poli L., Novelli L., Berloco P., Ferretti G., Iappelli M., Cortesini R., Novelli G. (2002). MARS (Molecular Adsorbent Recirculating System): Experience in 34 cases of acute liver failure. Liver.

[6] Isha S., Jenkins A.S., Hanson A.J., Satashia P.H., Narra S.A., Mundhra G.D., Hasan M.M., Donepudi A., Giri A., Johnson P.W. (2023). The Effect of Molecular Adsorbent Recirculating System in Patients with Liver Failure: A Case Series of 44 Patients. Transpl. Proc..

